# Mining Mammalian Transcript Data for Functional Long Non-Coding RNAs

**DOI:** 10.1371/journal.pone.0010316

**Published:** 2010-04-23

**Authors:** Amit N. Khachane, Paul M. Harrison

**Affiliations:** Department of Biology, McGill University, Montreal, Quebec, Canada; National University of Ireland Galway, Ireland

## Abstract

**Background:**

The role of long non-coding RNAs (lncRNAs) in controlling gene expression has garnered increased interest in recent years. Sequencing projects, such as Fantom3 for mouse and H-InvDB for human, have generated abundant data on transcribed components of mammalian cells, the majority of which appear not to be protein-coding. However, much of the non-protein-coding transcriptome could merely be a consequence of ‘transcription noise’. It is therefore essential to use bioinformatic approaches to identify the likely functional candidates in a high throughput manner.

**Principal Findings:**

We derived a scheme for classifying and annotating likely functional lncRNAs in mammals. Using the available experimental full-length cDNA data sets for human and mouse, we identified 78 lncRNAs that are either syntenically conserved between human and mouse, or that originate from the same protein-coding genes. Of these, 11 have significant sequence homology. We found that these lncRNAs exhibit: (i) patterns of codon substitution typical of non-coding transcripts; (ii) preservation of sequences in distant mammals such as dog and cow, (iii) significant sequence conservation relative to their corresponding flanking regions (in 50% cases, flanking regions do not have homology at all; and in the remaining, the degree of conservation is significantly less); (iv) existence mostly as single-exon forms (8/11); and, (v) presence of conserved and stable secondary structure motifs within them. We further identified orthologous protein-coding genes that are contributing to the pool of lncRNAs; of which, genes implicated in carcinogenesis are significantly over-represented.

**Conclusion:**

Our comparative mammalian genomics approach coupled with evolutionary analysis identified a small population of conserved long non-protein-coding RNAs (lncRNAs) that are potentially functional across *Mammalia*. Additionally, our analysis indicates that amongst the orthologous protein-coding genes that produce lncRNAs, those implicated in cancer pathogenesis are significantly over-represented, suggesting that these lncRNAs could play an important role in cancer pathomechanisms.

## Introduction

With the rapid development in high-throughput sequencing methods, one is now able to describe the mammalian transcriptome in great detail [1], [2], [3]. Not only is the mammalian transcriptome vast (comprising millions of RNA transcripts) [1], but also is quite unexpectedly diverse. For example, transcript lengths vary from 18 nucleotides (small interfering RNAs) to more than 15,000 nucleotides (in the case of macroRNAs or long non-protein-coding RNAs). Some protein-coding genes not only encode proteins but also contribute to the non-protein-coding RNA pool [4]. It is to be noted however that a significant proportion of the mammalian transcriptome could simply be ‘transcriptional noise’ [5], [6], [7], [8]. A wealth of data is now available for the two most studied mammalian genomes (human and mouse), and the chief challenge is to mine this data effectively for functionally relevant sequences. In this study, we have mined the full-length mammalian transcript (cDNA) data sets from the H-Invitational [3] and Fantom3 [2] projects, to identify potentially functional long non-protein-coding RNAs (lncRNAs). Our rationale was that those lncRNAs (> = 200 nucletoides) that are expressed in human and mouse and preserved in distant relatives, plus that show features of primary sequence and secondary structure conservation, are likely to be functional. We were also interested in knowing whether lncRNAs are transcribed from orthologous protein-coding genes, and if so, from which ones. A positive finding would indicate the conserved role of such protein-coding genes in producing noncoding RNAs, and also would indicate probable functional categories of the lncRNAs.

Previously, we developed a computational pipeline to annotate ‘transcribed pseudogenes’ (tψg), a class of long non-protein-coding RNAs that are homologous to protein-coding gene transcripts, but which harbor features indicative of a lack of protein-coding ability [9]. We discovered thousands of cases of transcribed pseudogene annotations in the human genome, and filtered the list to identify potential functional cases. In this paper, in a complementary analysis, we have identified conserved non-tψg members of the long non-protein-coding RNA category.

Long non-protein-coding RNAs (also termed ‘messenger-like’ or ‘messenger-RNA-like’ non-coding RNAs) usually bear features of mRNAs, *viz.*, 5′ capping, splicing and polyadenylation. However, they do not code for any protein. Although some well-characterized cases lack sequence conservation indicative of possible lineage-specific adaptive evolution [5], [8], a recent experimental work using chromatin immunoprecipitation and massively parallel sequencing (ChIP-Seq) identified several (>1500) ‘large, intervening ncRNAs’ that have some signatures of evolutionary conservation [10], thus challenging the current notion that lncRNA are not generally evolutionary conserved.

Examples of well-known functional long non-protein-coding RNAs include: *Xist*, and *H19*. *Xist* mediates X chromosome silencing as part of heterogametic dosage compensation during development [11], [12]. *H19* regulates expression of its neighboring gene *Igf2*, during embryogenesis, and may act as a tumour suppressor [13], [14], [15]. Recently, by means of comparative genomics, conserved long non-protein-coding RNAs have been identified [16], but authors have either ignored the regions that overlap protein-coding genes, or considered smaller length human transcripts (EST sequences) as a proxy for transcription in the absence of full-length non-protein-coding transcripts. It is possible that non-protein-coding sequences arise in part from protein-coding genes, for example, comprising of only UTR regions, or including retained introns, in their non-protein-coding transcripts. We propose that such cases have to be included in the category of long non-protein-coding RNAs, and that some cases cannot be clearly classified as either alternative splicing or partially overlapping lncRNAs. Another parameter we considered as essential was the length of potential lncRNA transcripts. In the present analysis, we used a lower bound of 200 nucleotides for the operational definition of lncRNAs, as in earlier work [8], [17], [18]. This criterion was chosen on the basis of a suitable practical cut-off during RNA purification steps to exclude small RNAs.

## Results and Discussion

### Identification of conserved and expressed lncRNAs

**Figure 1:**
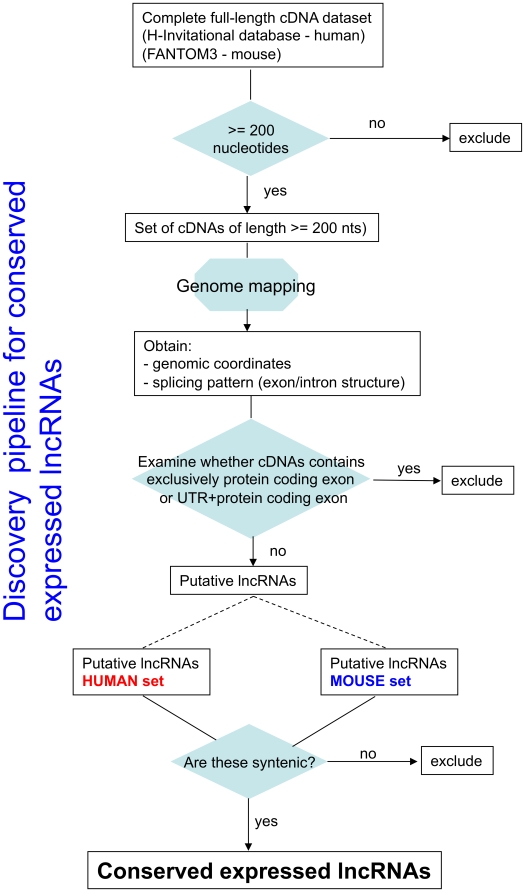
A schematic representation of the discovery pipeline for conserved expressed long non-protein-coding RNAs (lncRNAs).

H-Inv and Fantom3 projects catering to the human and mouse genomes, respectively, have generated thousands of sequence reads constituting expressed complements of the genomes [1], [2], [3]. Mere expression however does not necessarily indicate functionality. Many of these transcripts may simply be ‘transcriptional noise’ [5], [6]. Expressed elements that are syntenically conserved in phylogenetically divergent mammals are likely to be functional across *Mammalia*. Although a lot of transcripts could potentially be degradation products of UTRs or incompletely processed hnRNA fragments [7], natural selection would ensure preservation of biologically relevant genomic elements over millions years of evolution. Therefore, we developed a pipeline to identify potentially functional lncRNA candidates (fig. 1). We defined putative lncRNAs as full-length transcripts > = 200 nucleotides that do not: (i) exclusively contain known protein-coding exons; (ii) contain UTR plus protein-coding exons. We examined for syntenic conservation between the human and mouse genomes (see *Methods* for details). Additionally, we were also interested in identifying lncRNAs that originate from orthologous genes. Such genes may give hints to the function of lncRNAs. We found that 78 lncRNAs are syntenically conserved or originate from orthologous genes (Table 1). Some of these have detectable sequence similarity (Table 2). It is imperative that we find previously characterized functional lncRNAs in the list. Indeed, our list contains two well-documented examples of lncRNAs, namely, *H19* [13], [14] and *Xist* [11], [12]. We also looked for lncRNA candidates that could have arisen due to internal priming as described by Nordstrom *et al.* [7] and Nam *et al.* [19]. For this a 50 bp genomic region downstream to the identified putative human lncRNAs was examined for the presence of poly(A) rich region. We found that only 3 out of the 78 putative lncRNAs may have arisen due to internal priming, thus indicating that the majority of the identified lncRNAs in this study are likely to be genuine candidates.

**Table 1:** General statistics for the 78 conserved lncRNAs.

Category	Number of cases*
Syntenically conserved	With significant sequence homology: 11
	Without significant sequence homology: 67
Genomic location	Protein-coding region: 57
	Non-protein-coding region: 21
Spliced forms	Spliced: 64
	Non spliced: 14

*BLAST e-value set was to <1×10^−6^. The protein-coding region annotations were taken from the ENSEMBL website (www.ensembl.org).

**Table 2:** A summary of the analysis results for preservation, sequence conservation and occurrence of secondary structure motifs in mouse lncRNAs that have orthologous human counterparts (with BLAST homology).

Fantom3 entries	H-Inv db entries (syntenic to mouse lncRNA and having BLAST homology, e-value: <0.01)	Preservation in other mammals*	Sequence identities between conserved lncRNAs and between orthologous flanking regions indicated in brackets	Conservation of secondary structure motifs
A230108N10	HIT000394689.1	HMDC	80.3% (not conserved)	no
D730031O06	HIT000294155.8	HMDC	64.3% (not conserved)	yes
A430070C22	HIT000091723.8	HMDC	10.12% (not conserved)	no
1600017P15	HIT000389575.3	HM	72% (not conserved)	no
A130061G12	HIT000294554.8	HMDC	25.8% (34.8%)	yes
2600002C05	HIT000323535.8	HMDC	43.4% (not conserved)	yes
9530073M10	HIT000093538.10	HMDC	66.1% (40.15%)	no
5430433I11	HIT000282711.8	HMDC	87.9% (17.2%)	yes
5330421F07	HIT000248175.9	HMDC	28.1% (7.2%)	yes
1110021C24	HIT000292834.10	HMDC	48.1% (11.3%)	no
G370125G16	HIT000430538.1	HMDC	39.4% (not conserved)	no

*‘H’ refers to human, ‘M’ to mouse, ‘D’ to dog and ‘C’ to cow.

Note: For the calculation of sequence conservation, orthologous sequences to mouse lncRNAs were identified in the human genome using synteny maps and BLAST searches (e-value<0.01) and subjected to further evolutionary analysis.

### Origin of lncRNAs from various genomic positions

**Figure 2:**
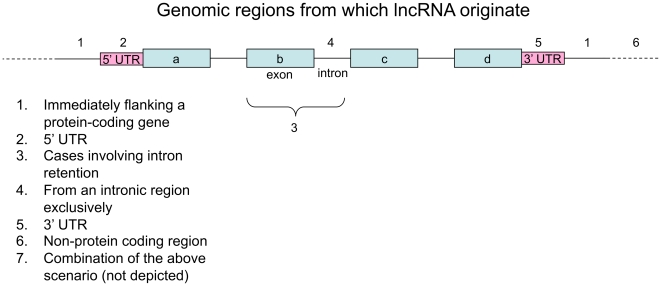
A schematic representation of different genomic regions from which lncRNA originate relative to the structure of a protein-coding gene.

Next, we analyzed the various genomic segments that participate in the generation of these lncRNAs. We found that the above shortlisted lncRNAs are predominantly (70 out of 78 cases) derived from protein-coding genes (including intronic regions) or lay directly beside them (<1000 nts distance). This suggests that the lncRNAs depend on the same promoter regions for transcription, as the nearby protein-coding genes. Interestingly, 18 lncRNAs are expressed from UTRs, exclusively. Nineteen of them originate from introns, while others arise from a combination of different categories of genomic DNA, as exemplified in fig. 2. The lncRNAs that originate from UTRs may have a possible regulatory role akin to the role of specific UTRs as riboswitches [20].

### A significant proportion of putative functional lncRNAs originate from cancer-related genes

We found that ∼35% (20/57) of the protein-coding genes that overlap with the annotations of the identified lncRNAs are implicated in the causation of diseases, particularly cancer (Table 3). To assess for the possible enrichment of such genes we proceeded as follows. We counted the number of lncRNA-producing genes from our list that are listed in the ‘CGMIM’ database [21]. ‘CGMIM’ provides a list of all gene entries in OMIM that referred to some type of cancer. ∼18% of the protein coding genes that produce lncRNAs (10/57) have reference to cancer (see Table 3), whereas only 9% of all human protein coding genes (2147/23621) have reference to cancer. The above difference is statistically significant (chi-square test, *P-value*: 0.047; hypergeometric probability *P-value* = 0.018), suggesting that the genes implicated in cancer causation have a higher tendency to produce lncRNAs. It has been earlier found that ncRNAs have altered expression/splicing in cancer cells [22], [23]. Thus, we believe that the identified lncRNAs could have potential roles in oncogenesis, although of course, we cannot ascertain here whether there is a ‘cause-and-effect’ relationship.

**Table 3:** List of lncRNAs associated with known genes implicated in cancer pathogenesis.

H-Inv id	Gene name	Cancer	References
HIT000067299.10	*brf1*	Lymphoma	CGMIM
HIT000064387.8	*cbfa2t2*	Leukemia	CGMIM
HIT000257890.10	*dicer1*	Breast, Melanoma, Ovarian	CGMIM, [25]
HIT000277951.8	*eif4g2*	Leukemia	CGMIM, [36]
HIT000323535.8	*hnrpdl*	Leukemia	CGMIM
HIT000389429.2	*ppard*	Colorectal	CGMIM, [37], [38]
HIT000327147.7	*slc12a2*	Colorectal	CGMIM
HIT000079026.8	*st7*	Brain, Breast, Colorectal, Prostrate, Ovarian	CGMIM, [28], [39]
HIT000067550.9	*st8sia1*	Brain, Melanoma	CGMIM
HIT000276030.9	*xist*	Breast, Ovarian	CGMIM
HIT000284226.9	*rab4A*	Oncogene	OMIM
HIT000024195.13	*akt3*	Melanoma	[40], [41], [42], [43]
HIT000383650.1	*ptch1*	Basal cell carcinoma	OMIM
HIT000243731.8	*rab18*	reduced expression in Pituitary tumors and its overexpression reverts growth hormone hypersecretion	[44]
HIT000248175.9	*nav2/Helad1*	Colorectal carcinomas	[45]
HIT000075518.7	*rerg*	Breast cancer	[46]
HIT000071420.7	*dach1*	Prostate cancer, Breast cancer	[47], [48]
HIT000389219.2	*rad51L1*	Pulmonary chondroid hamartoma, Uterine leiomyomas	[49], [50]
HIT000089413.9	*tnfaip2*	Acute promyelocytic leukemia	[51]
HIT000330125.5	*nat1*	Non Hodgkin lymphoma, Urinary bladder cancer susceptibility, Colorectal adenoma susceptibility	[52], [53], [54]

Note: ‘CGMIM’ database is accessible at http://www.bccrc.ca/ccr/CGMIM/ and ‘OMIM’ database at www.ncbi.nlm.nih.gov/omim/.

### Putative functional lncRNAs typically bear single non-coding exon

We performed an intron/exon analysis on the identified set of putative functional lncRNAs to study the contribution of splicing to their generation, thereby assessing the possible relationship between lncRNA splicing and function. We found that a vast majority (∼83%, 65 out of 78) of the above lncRNAs contains just a single exon. This suggests that functional lncRNAs tend to have a single exon, and may thus (although speculative) reflect avoidance of unnecessary (complex) involvement of splicing mechanism regulation in lncRNA generation.

Examples of potential functional conserved lncRNAs include cases that overlap Dicer and U2AF2. *Dicer* is an endoribonuclease that cleaves double-stranded RNAs into shorter double-stranded segments called small interfering RNAs (siRNAs) [24], [25], [26]. The *U2AF2* gene encodes the U2 snRNP auxiliary factor, which participates in splicesome assembly formation by binding to polypyrimidine tracts [27].

### Role of some lncRNAs in post-transcriptional regulation

**Figure 3:**
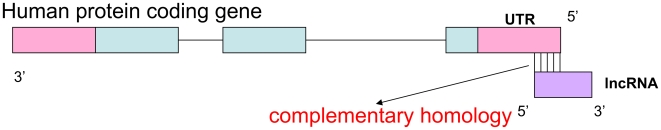
A model for antisense regulation of target mRNA transcripts by lncRNAs. The following lncRNA sequences: HIT000079026.8 and HIT000091723.8, have complementary relationship to UTR of the following protein-coding transcripts: ENST00000393449 and ENST00000383790, respectively.

Long non-protein-coding RNAs are known to play a role in the post-transcriptional regulation of target genes [8]. We found two examples of lncRNAs (HIT000079026.8 and HIT000091723.8) that are transcribed in the antisense direction to the orientation of the UTR region of the protein-coding gene (in these cases, also, there are no other protein-coding exons that overlap on the other strand in these particular genomic regions). These lncRNAs could therefore act as negative regulators of gene expression by complementary binding to the UTRs of target mRNAs (fig. 3). A good example is that of an lncRNA associated with the *ST7* gene. Functional analyses have revealed that *ST7*, a tumor-suppressor gene, plays a role in the development of certain cancer types [28]. Therefore, it is possible that the lncRNA may also be involved in carcinogenesis. Based on the above findings, we suggest a general model for negative feedback post-transcriptional regulation (fig. 3) of gene transcript effectuated *via* complementary hybridization between UTR-derived lncRNAs and parent mRNAs. An experimental validation, however, is necessary.

### Evidence for selection on the identified putative functional lncRNAs

We analysed for features of selection in orthologous lncRNAs that have detectable (significant) similarity between them. As lncRNAs from mouse and human do not completely overlap although they show significant homology, we used mouse lncRNAs as reference sequences, and deduced the orthologous human counterpart by BLASTing [29] mouse lncRNAs against the human genome. We compared the sequence identities of these deduced orthologous lncRNAs to their flanking regions. Buffer (intergenic) regions flanking mouse lncRNAs, of length equivalent to that of lncRNA, were selected and examined for the presence of similar counterparts in near syntenic locations in other mammals. These were then aligned using a global alignment algorithm [30]. From the results (Table 2), it is clear that many lncRNAs do not have conserved flanking regions or are not as significantly conserved as lncRNAs. This indicates that the identified lncRNAs are under selection, thus giving further support to their potential functionality.

### Secondary structure analysis

We then investigated whether any of the long non-coding RNAs (> = 200 nts) encode thermodynamically stable and conserved secondary-structure motifs, a finding that could lend support to their functional role. For this, we used the program RNAz [31] to examine for the conservation of stable secondary structure motifs in orthologous sequences. RNAz calculates a “RNA class probability” or P-value based on structural conservation index and thermodynamic stability scores. Alignments with P>0.5 are classified as functional RNA. We found that nearly 45% of the identified lncRNAs, *i.e.*, 5 of the 11 orthologous lncRNAs that have detectable homology, have conserved and stable secondary structure motifs (*i.e.*, P-value>0.5). This further strengthens our case that these lncRNAs could represent biologically relevant sequences.

### Genomic conservation in other mammals

Expression *per se* does not indicate functionality. Sequences of long noncoding RNAs that are present in distantly related mammals (non-coding RNA orthologs) indicate the presence of evolutionary pressure for their preservation. Such preservation indicates possible functionality. Out of the 11 in our list, we find that 9 are conserved in human, mouse, dog and cow. One of them is preserved in human, mouse and dog, whereas the remaining one is preserved only in human and mouse. This indicates that the identified lncRNAs have been conserved across mammalian speciation.

### Evolutionary analysis of codon substitution rates

**Figure 4:**
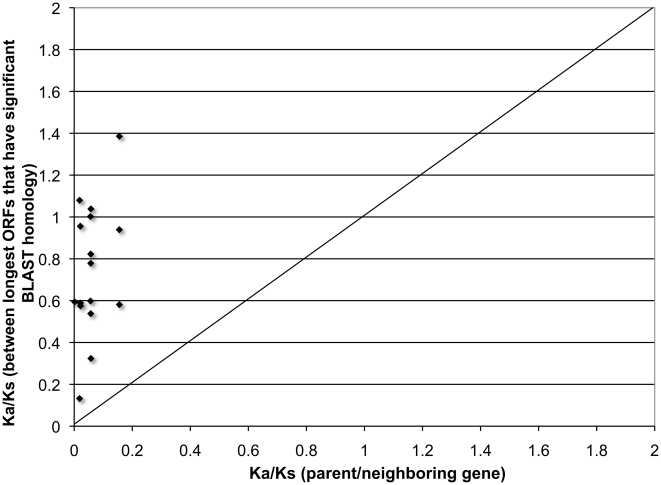
Assessment for protein-coding ability. Comparison between Ka/Ks values of long ORFs derived from six-frame conceptual translation for human-mouse lncRNAs and orthologous neighboring protein-coding genes.

A measure of selection pressure for protein-coding ability of genes is the ratio of non-synonymous to synonymous substitution rates (Ka/Ks). Values significantly ≪1.0 indicate purifying selection, whereas neutral selection theoretically yields a value of ∼1.0. We compared Ka/Ks values for the above 11 lncRNA ortholog pairs (termed Ka/Ks*_lncRNA-ortho_*) with the corresponding Ka/Ks values for their parent/nearby genes (Ka/Ks*_parent-ortho_*) (fig. 4). These Ka/Ks values were calculated for the longest ORFs from each lncRNA. Only 19 ORFs out of the 66 possible longest ORFs obtained following six-frame conceptual-translations, were found to have significant similarity to respective human counterparts. Although we considered best-case similarity between any two conceptually translated long open reading frames (see Materials and Methods), we found that codon substitution patterns do not support the hypothesis of protein-coding ability, as the Ka/Ks ratios for these alignments are mostly in the range 0.5–1.5.

### Conclusion

In this comparative study, we mined publicly available (experimental) data sets of mammalian full-length cDNAs for evolutionarily conserved lncRNAs. These represent novel genomic elements of likely functional relevance. Of course, it cannot be ruled out that some of these apparent lncRNAs are conserved to produce functional short peptides, such as was recently described for two mRNAs in Drosophila [32]. Because quite a number of lncRNAs arise from protein-coding regions, it is conceivable that they are involved in functional roles complementing to that of the parent protein-coding gene. In this vein, we have found that cancer-related genes are over-represented in the protein-coding genes that are contributing to the pool of lncRNAs. This therefore suggests that lncRNAs may play an important role in cancer pathomechanisms.

## Materials and Methods

### Collection of data

Full-length cDNA datasets for human and mouse were obtained from the H-InvDB (www.h-invitational.jp/) and Fantom3 (http://fantom3.gsc.riken.jp/) databases respectively. Complete genome sequences of mammals were obtained from http://www.ensembl.org (Ensembl release 47 for human genome; Ensembl release 48 for other mammals, namely, rhesus monkey, mouse, rat, cow and dog). Full-length cDNAs with length> = 200 nucleotides only were considered for further analysis, as analysis of small RNAs was not the focus of this study. To identify genomic locations of transcripts in mammals, cDNAs were mapped onto the respective genome using GMAP software [33] with match criteria of ≥99% sequence identity and ≥99% sequence coverage.

### Identification of orthologous lncRNAs in various sequenced mammalian genomes

Orthologous counterparts to mouse lncRNAs are detected by the presence of a similar sequence at the syntenic position in the other mammalian genome. Based on this criterion, a search was carried out in the target mammal as indicated in the synteny maps, to locate orthologous lncRNAs. The following mammals were included in the analysis: human, monkey, mouse, rat, cow and dog. The pair-wise synteny map data for the various mammals were obtained from http://genome.ucsc.edu/. For a schematic representation of the discovery pipeline for putative functional ncRNAs, see fig. 1.

### Ka/Ks calculation

Although orthologous lncRNAs from mouse and human show significant similarity, they however do not completely overlap. Hence, we deduced the orthologous human lncRNAs counterpart by BLASTing [29] mouse lncRNAs against the human genome. Next, putative lncRNA sequences were conceptually translated in all six frames, and the longest ORF in each frame translation was identified. These long ORFs were then pairwise aligned to assess for possible homology at the protein sequence level using BlastP program of BLAST package [29]. Those showing significant pairwise BlastP homology were short-listed and were used for the calculation of Ka/Ks values using the PAL2NAL web server (www.bork.embl.de/pal2nal/), which integrates PAL2NAL tool [34] and the PAML 4 software package [35].

### Secondary structure prediction

RNAz predicts structurally conserved and thermodynamically stable secondary structures (http://rna.tbi.univie.ac.at/cgi-bin/RNAz.cgi). We used the RNAz program with default parameters to check for conserved secondary structure motifs in the set of human-mouse lncRNA orthologs.
